# Effects of glutathione depletion on the cytotoxicity of agents toward a human colonic tumour cell line.

**DOI:** 10.1038/bjc.1987.127

**Published:** 1987-06

**Authors:** J. Jordan, M. d'Arcy Doherty, G. M. Cohen

## Abstract

Levels of glutathione (GSH) in tumour tissue may be important in determining the clinical response to certain anticancer agents. Recent reports have suggested that D,L-buthionine-S,R-sulphoximine (BSO), a specific inhibitor of GSH synthesis, may be used to deplete tumour cell GSH and thus increase the therapeutic ratio of these agents. We have previously shown that 1-naphthol is a potential antitumour agent, and that its possible metabolite 1,4-naphthoquinone is thiol reactive and capable of redox cycling. It was therefore of interest to investigate the effect of pretreatment with BSO, on the toxicity of these agents, to tumour cells. For comparison we included three other cytotoxic agents, melphalan, helenalin and menadione, the toxicities of which are reported to be modulated by intracellular GSH. Depletion of GSH using BSO did not effect the toxicity of 1-naphthol, or 1,4-NQ but did produce slight potentiation of the cytotoxicities of menadione, helanalin and melphalan. The lack of effect of BSO on 1-naphthol and 1,4-NQ is not easily explained but if one also considers the modest potentiation of cytotoxicity+ achieved with the other agents studied, the potential use of BSO in combined chemotherapy is at best rather modest.


*Br. J. Cancer* (1987), **55**, 627–631 © The Macmillan Press Ltd., 1987

### Effects of glutathione depletion on the cytotoxicity of agents toward a human colonic tumour cell line

J. Jordan, M. d'Arcy Doherty & G.M. Cohen

*Toxicology Unit, Department of Pharmacology, The School of Pharmacy, University of London, 29/39 Brunswick Square, London WC1N 1AX, UK.*

**Summary** Levels of glutathione (GSH) in tumour tissue may be important in determining the clinical response to certain anticancer agents. Recent reports have suggested that D,L-buthionine-S,R-sulphoximine (BSO), a specific inhibitor of GSH synthesis, may be used to deplete tumour cell GSH and thus increase the therapeutic ratio of these agents. We have previously shown that 1-naphthol is a potential antitumour agent, and that its possible metabolite 1,4-naphthoquinone is thiol reactive and capable of redox cycling. It was therefore of interest to investigate the effect of pretreatment with BSO, on the toxicity of these agents, to tumour cells. For comparison we included three other cytotoxic agents, melphalan, helenalin and menadione, the toxicities of which are reported to be modulated by intracellular GSH. Depletion of GSH using BSO did not effect the toxicity of 1-naphthol, or 1,4-NQ but did produce slight potentiation of the cytotoxicities of menadione, helanalin and melphalan. The lack of effect of BSO on 1-naphthol and 1,4-NQ is not easily explained but if one also considers the modest potentiation of cytotoxicity achieved with the other agents studied, the potential use of BSO in combined chemotherapy is at best rather modest.

Based on our findings that 1-naphthol is selectively toxic to short term organ cultures of human colonic tumour tissue compared to normal colonic tissue from the same patients, we suggested the potential use of 1-naphthol or related compounds in cancer chemotherapy (Cohen *et al.*, 1983; Wilson *et al.*, 1985). Recently we have also shown an antitumour activity of 1-naphthol against Ehrlich ascites tumour cells (Jones *et al.*, 1987) and it therefore is of interest to elucidate its mechanism of toxicity and formation of possible reactive metabolites. 1-Naphthol may be metabolised by a microsomal mixed function oxidase to cytotoxic naphthoquinones, primarily 1,4-naphthoquinone (d'Arcy Doherty *et al.*, 1984*a,b*, 1985). The toxicity of both 1-naphthol and its possible metabolites 1,2-naphthoquinone and 1,4-naphthoquinone, to isolated hepatocytes, is prececed by a rapid depletion of intracellular glutathione (GSH) (d'Arcy Doherty *et al.*, 1984*b*).

GSH is the major nonprotein thiol in the cell and plays a critical role in cellular defences against oxidative stress, free radicals and alkylating agents (Meister & Anderson, 1979). One of the problems associated with chemotherapy is the wide range of sensitivities to treatment with any or one agent, which is thought to be, in part, due to the differences in sulphydryl levels in tumours. Several recent reports, have therefore considered the potential of modulating intracellular GSH levels in order to increase the chemotherapeutic efficacy of certain antitumour agents, whose toxicity is modulated by GSH (Akman *et al.*, 1985; Arrick *et al.*, 1983; Capranico *et al.*, 1986; Hamilton *et al.*, 1985; Russo *et al.*, 1986; Suzukake *et al.*, 1982, 1983).

The cytotoxic and antitumour effects of certain quinones e.g. adriamycin and menadione, are thought to be related to oxidative stress which arises through the capacity of those compounds to redox cycle (Kappus & Sies, 1981; Thor *et al.*, 1982). Flavoenzymes catalyze a one electron reduction of naphthoquinones to form semiquinone radicals which can readily autoxidise in the presence of molecular oxygen ($O_2$) forming large amounts of the superoxide anion radical ($O_2^{\cdot -}$), which may then in turn spontaneously, or in a reaction catalysed by superoxide dismutase, dismutate to produce hydrogen peroxide ($H_2O_2$) which in turn may undergo a metal catalysed reaction forming hydroxyl radical ($OH^{\cdot}$), an extremely powerful oxidant (Bachur *et al.*, 1978; Thor *et al.*, 1982; Wendel *et al.*, 1981; Powis *et al.*, 1981). This may lead to conditions of oxidative stress, lipid peroxidation, damage to DNA and other vital cellular constituents (Smith *et al.*, 1985). GSH may protect against naphthoquinone mediated oxidative stress in several ways, including direct reaction with the parent naphthoquinone or its semiquinone radicals, or by removing with glutathione peroxidase either $H_2O_2$ formed or hydroperoxides produced as a result of lipid peroxidation (Nickerson *et al.*, 1963; Wendel *et al.*, 1981). It seems reasonable therefore, to suggest that GSH may play a role in the protection of tumour cells against 1-naphthol or its possible metabolite, 1,4-naphthoquinone. In this study, intracellular GSH was depleted in LoVo cells, a human colonic adenocarcinoma cell line (Drewinko *et al.*, 1976), using DL-buthionine-S-R sulphoximine (BSO), a specific inhibitor of γ-glutamyl cysteine synthetase, the rate limiting enzyme in GSH synthesis (Griffith & Meister, 1979), and the effect on the toxicity of 1-naphthol and its possible metabolite 1,4-naphthoquinone were studied. For comparison, we included menadione (2-methyl-1,4-naphthoquinone) and two alkylating agents, melphalan and helenalin. The chemosensitivity of melphalan and helenalin has previously been shown to be increased in the presence of BSO. Tumour cell lines resistant to the alkylating agent melphalan were found to have elevated GSH and GSH S-transferase levels and sensitivity was restored using BSO, to deplete GSH, in such cell lines (Green *et al.*, 1984; Hamilton *et al.*, 1985). BSO has also been shown to augment the lysis of tumour cells by helenalin, therefore this agent was included as a positive control (Arrick *et al.*, 1983). Menadione, a derivative of vitamin K has been extensively studied, with regard to quinone toxicity and its reactions with GSH (Thor *et al.*, 1982) and is currently undergoing clinical trial with the antimetabolite 5-fluorouracil (Chlebowski *et al.*, 1983).

Depletion of GSH using BSO did not effect the toxicity of 1-naphthol or 1,4-naphthoquinone, as assessed by two end points of toxicity. However moderate potentiation was observed with helenalin, melphalan and menadione. As the effect of BSO on the cytotoxicity of all these agents was not dramatic – the potential use of BSO in combined therapy in the clinic may be limited.

### Materials and methods

### *Cell Culture*

LoVo human carcinoma cells (Drewinko *et al.*, 1976)

Correspondence: G.M. Cohen.
Received 10 November 1986; and in revised form, 16 February 1987.

supplied by Dr Bridget Hill, ICRF London, were grown in monolayer culture in Hams F-12 medium supplemented with 10% foetal calf serum (Flow Labs), 1 mM L-glutamine, penicillin 100 u ml$^{-1}$ and streptomycin 100 $\mu$g ml$^{-1}$ (Gibco). The cells were maintained at 37°C in a humidified atmosphere of 5% $CO_2$ and routinely subcultured each week. Cells in exponential growth phase were used in all experiments.

*Drug exposure and cytotoxicity*

For assay of protein synthesis inhibition, 200 $\mu$l aliquots of a cell suspension of density $5 \times 10^4$ cells ml$^{-1}$, were seeded into 96 wells of a flat-bottomed microtitre plate. The ability of agents to inhibit $^3$H-leucine incorporation into protein was carried out as previously described (Wilson *et al.,* 1985).

For cell growth determinations, 1.5 ml of a $1.4 \times 10^4$ cells ml$^{-1}$ suspension of cells were seeded into 3 cm diameter petriplates. After incubation overnight at 37°C, 5% $CO_2$, the medium was removed and replaced with complete drug-free medium or medium containing 0.2 mM BSO and again incubated overnight (18 h). Cells were then exposed for 5 h to the cytotoxic agents in fresh medium in the presence or absence of 0.2 mM BSO, as before. After 5 h the medium was then removed by aspiration, replaced with fresh medium and incubated for a further 48 h at 37°C, 5% $CO_2$, and the cell number estimated by counting an aliquot of trypsinised cells with a Coulter Counter. All experiments were repeated 3–6 times and standard errors calculated.

*GSH determination*

*In cultured cells* Monolayer cultures in 3 cm dishes were washed twice with 0.9% saline and the GSH extracted with 6.5% TCA at 4°C for 10 min. GSH was assayed by the method of Hissin and Hilf (1976) using o-phthalaldehyde (o-PT) and results expressed as nmol GSH $10^{-6}$ cells.

*Following chemical reactions of test agents with GSH* Various concentrations of the chemicals were incubated at 37°C in HEPES (10 mM) (pH 7.5) with 100 $\mu$M GSH and the GSH remaining determined using o-P as before.

### Results

*Effect of BSO on tumour cell GSH*

BSO (0.05–0.2 mM) caused a time dependent depletion of GSH (Figure 1) from a starting level of 6.6 ± 0.7 nmol GSH $10^{-6}$ cells present in control cells. After 24 h exposure to BSO (0.2 mM), a maximum depletion to 12% of control was obtained with viabilities of 98% ± 2.1 and 95.4 ± 8.9% as assessed by protein synthesis inhibition and cell numbers, respectively. A concentration of BSO (0.2 mM) was therefore chosen for overnight incubation (18 h) to deplete GSH.

**Figure 1** Time course of GSH depletion in LoVo cells, following exposure to BSO. Results are expressed as a percentage of GSH present in untreated cells. (●——●) 0.05 mM; (▲----▲) 0.1 mM and (○----○) 0.2 mM BSO.

*Effect of GSH modulation on the cytotoxicity of the chemicals to LoVo cells*

After exposure of LoVo cells to 1-naphthol for 5 h, protein synthesis inhibition was a more sensitive indicator of cytotoxicity than cell numbers, 48 h after exposure (Table I). The opposite effect was observed with melphalan, when the $IC_{50}$ values obtained using cell numbers was one fifth that determined by protein synthesis inhibition (Table I). For all

**Table I** Effect of BSO on the cytotoxicity of the chemicals to LoVo cells

| | $IC_{50}$ $\mu$M (using $^3$H-leucine) | | | $IC_{50}$ $\mu$M (using Cell Number) | | |
|---|---|---|---|---|---|---|
| *Chemical* | *−BSO* | *+BSO* | *DMF*[a] | *+BSO* | *−BSO* | *DMF*[a] |
| 1-Naphthol | 543 ± 34 | 540 ± 41 | 1 | 950 ± 134 | 960 ± 154 | 1 |
| 1,4-NQ | 13 ± 1.5 | 13 ± 1.5 | 1 | 25.5 ± 6.2 | 24.5 ± 6.3 | 1 |
| Menadione | 30.4 ± 3.3 | 23.8 ± 4.8 | 1.27[b] | 24.6 ± 6.2 | 19.5 ± 5.6 | 1.8[b] |
| Helenalin | 3.80 ± 0.2 | 1.53 ± 0.7 | 2.48[b] | 2.6 ± 0.9 | 1.07 ± 0.6 | 2.4[b] |
| Melphalan[c] | 57.8 ± 5.6 | 43 ± 11 | 1.38[b] | 12.7 ± 3.8 | 6.7 ± 1.7 | 1.9 |

[a]DMF = Dose modification factor; [b]Significant difference ($P < 0.05$) between $IC_{50}$ values ±BSO, using a paired *t*-test; [c]Significant difference ($P < 0.05$) between $IC_{50}$ values determined by the two criteria of assessing cytotoxicity, using a paired *t*-test. Each experiment was repeated at least three times and a minimum of four wells per concentration was used in every experiment.

other agents used in this study i.e. menadione, helenalin, and 1,4-NQ, no significant difference in the $IC_{50}$ values, as assessed by either criteria were observed (Table I).

Depletion of GSH using BSO, did not effect the cytotoxicity of 1-naphthol, or 1,4-naphthoquinone as assessed by either method of toxicity, whereas the cytotoxicities as assessed by inhibition of protein synthesis, of menadione, helenalin and melphalan were significantly potentiated due to pretreatment and incubation with BSO (Table I). BSO treatment also potentiated the cytotoxicity of menadione and helenalin, as assessed by cell numbers. A small but not significant effect was observed with melphalan. The maximum modification of an $IC_{50}$ value in the presence of BSO was a 2.5 fold decrease in the helenalin $IC_{50}$ value (Figure 2), all other effects were less than 2 fold.

**Figure 2** Effect of BSO on the toxicity of helenalin to LoVo cells. (a) Protein synthesis was assessed by incorporation of $^{3}H$-leucine and (b) cells numbers were determined 48 h after drug exposure. *Significant at $P<0.05$ paired $t$-test.

*Effects of chemicals on tumour cell GSH*

LoVo cells were incubated with equitoxic concentrations (as assessed by protein synthesis inhibition after 5 h exposure) and the GSH levels determined over a 2 h exposure (Figure 3). Within 30 min, 1,4-NQ caused over 95% depletion of GSH in LoVo cells and this level was maintained over the 2 h exposure. Menadione also caused an extensive but insignificant, depletion due to the large variation in the response. Melphalan and 1-naphthol did not deplete GSH, however helenalin actually caused a small but not significant increase in GSH above control value at 30 min which returned to normal at 60 and 120 min (results not shown).

*Reactivity of chemicals with GSH in solution*

The chemicals were incubated with GSH in buffered solution (Figure 4). 1-Naphthol (100 μM–1 mM) did not react with GSH in solution whereas 1,4-NQ was highly reactive. After 15 min incubation with 100 μM 1,4-NQ, less than 10% of the GSH present at the start of the incubation (100 μM)

**Figure 3** Effect of equitoxic concentrations of cytotoxic chemicals on tumour cell GSH. The GSH levels after 30 min exposure are shown and the results are expressed as percentage GSH present in untreated cells. The concentrations of 1-naphthol, 1,4-NQ, menadione, melphalan and helenalin were 250, 10, 20, 20 and 1 μM respectively. *Significant $P<0.05$ unpaired $t$-test.

remained (Figure 4). Overall reactivity of the chemicals with GSH in solution followed the order 1,4-NQ > menadione > melphalan ≃ helenalin ≫ 1-naphthol.

### Discussion

Our results demonstrate that GSH depletion, using BSO to inhibit GSH synthesis, may affect the cytotoxicity of selected agents to LoVo cells growing *in vitro* (Table I). Cytotoxicity was assessed by inhibition of both protein synthesis and cell numbers. In a previous study with LoVo cells, little difference was observed in the toxicity of 1-naphthol or 1,4-NQ, when assessed either by inhibition of protein synthesis or by a clonogenic assay (Wilson *et al.*, 1985). The possibility that BSO or the drug treatments altered the uptake of [³H]-leucine or its pool sizes cannot be excluded. However in a similar study with human lung tumour cells, BSO did not affect the uptake of [¹⁴C]-leucine (Brodie & Reed, 1985). The cytotoxicity of 1-naphthol assessed by protein synthesis inhibition or cell number determination after 24 h, was not affected by BSO, indicating that GSH may not be involved in protecting LoVo cells from the toxicity of 1-naphthol. In addition, BSO did not affect the cytotoxicity of 1,4-NQ, a possible metabolite of 1-naphthol. Based on these results, the possible involvement of 1,4-NQ in

**Figure 4** Chemical interaction of agents with GSH in solution. Results are expressed as percentage GSH remaining after 15 min incubation at 37°C. *Significant at $P<0.05$ unpaired $t$-test.

**Table II** Effects of glutathione depletion on the cytotoxicity of antitumour agents

| *Treatment* | *Cell line* | *Measurement of toxicity* | *Effect* | *DMF*[a] (*if given*) | *Ref.* |
|---|---|---|---|---|---|
| Helenalin | P815 | 51 Cr | potentiation | 4.7 | Arrick *et al.* (1983) |
| Jatrophone | P815 | 51 Cr | potentiation | 21.3 | |
| Adriamycin | P815 | 51 Cr | none | | Arrick *et al.* (1983) |
| | ADR resistant | clonogenic | sensitisation | | Hamilton *et al.* (1985) |
| | V79 | clonogenic | potentiation | 4–10.5 | Russo *et al.* (1986) |
| | A549 | clonogenic | none | | Russo *et al.* (1986) |
| Daunorubicin | P388 | cell number | none | | Romine & Kessel |
| | P388/ADR resistant | | none | | (1986) |
| Bisthiosemi- | P388 | cell number | potentiation | 3.4 | Romine & Kessel |
| carbozone | P388/ADR | cell number | potentiation/ | 1.4 | (1986) |
| B2844 | resistant | | sensitisation | | |
| $H_2O_2$ | | | | | |
| —preformed | P815 | 51 Cr | none | | Arrick *et al.* (1982) |
| | endothelial | 51 Cr | potentiation | | Tsan *et al.* (1985) |
| —generated | P815 | 51 Cr | potentiation | 3.5 | Arrick *et al.* (1982) |
| | endothelial | 51 Cr | potentiation | | Tsan *et al.* (1982) |
| Melphalan (L-PAM) | A1847 LPAM resistant | clonogenic | sensitisation | 3.5–10 | Green *et al.* (1984) |
| | L1210 LPam resistant | clonogenic | sensitisation | | Somfai-Relle *et al.* (1984) |
| BCNU[b] | P815 | 51 Cr | none | 0.99 | Arrick *et al.* (1982) |
| Vinblastine | | 51 Cr | none | 1.17 | |
| Cytosine | | 51 Cr | none | 0.74 | |
| Arabinoside (Arac) | | | | 0.71 | |
| Maytansine | | 51 Cr | none | 0.91 | |
| Irradiation | lymphoid | trypan blue | potentiation | | Dethmers & |
| (DTNB)[c] | | exclusion | potentiation | | Meister (1981) |
| 5-Fluorouracil | | | none | | |
| Vincristine | | | none | | |
| Neocarzino- | V79 | clonogenic | protection | | DeGraff *et al.* (1985) |
| statin | CCL-210 (normal) | clonogenic | none | | Russo *et al.* (1986) |

[a]Dose modifying factor; [b]1,3-bis[2-chloroethyl]-1-nitrosourea; [c]5,5-dithiobis(2-nitrobenzoic acid).

the toxicity of 1-naphthol cannot be excluded. The lack of effect of BSO on the toxicity of both 1-naphthol and 1,4-NQ was rather surprising, as with isolated hepatocytes both these compounds caused a depletion of intracellular GSH prior to cell death (d'Arcy Doherty *et al.*, 1984*b*). However with LoVo cells, 1,4-NQ but not 1-naphthol, caused a depletion in GSH (Figure 2). This may be due to differences in the ability of these different cell types to activate these compounds or to deal with the accompanying oxidative stress.

It was of interest that under the same conditions, BSO caused a small but significant potentiation of menadione cytotoxicity but had no effect on the structurally related 1,4-NQ (Table I). One possible explanation for this difference is that the two quinones may exert their toxicity by different mechanisms due to the higher chemical reactivity of 1,4-NQ (Figure 4).

The cytotoxicities of the two alkylating agents in the study, helenalin and melphalan, were potentiated in the presence of BSO (Table I) in agreement with other studies (Arrick *et al.*, 1983; Green *et al.*, 1984; Hamilton *et al.*, 1985; Suzukake *et al.*, 1982). Of the four chemicals used in this study the greatest potentiation of cytotoxicity in the presence of BSO was exhibited by helenalin (Table I and Figure 2). GSH may protect against helenalin cytotoxicity by conjugation, prior to alkylation of target molecules, prevent cross linking or restore critical sulphydryl groups (Hall *et al.*, 1977, 1978). It is of interest to note that in the presence of helenalin (1–10 $\mu$M), LoVo cell GSH was not depleted, suggesting that modulation of GSH may be of importance in determining the toxicity of agents that do not deplete GSH.

Recently a number of studies have investigated the possible relationship between resistance to melphalan (L-PAM mustard), a bifunctional alkylating agent and thiol status of the cells (Suzukake *et al.*, 1983). In this study, a modest potentiation of melphalan toxicity, to LoVo cells was observed in the presence of BSO suggesting a protective role for GSH against the cytotoxic action of melphalan, probably via conjugation reactions. This potentiation of melphalan toxicity was less than that observed by others (Table II) and may be due to a number of possibilities such as the different measures of cytotoxicity used or to lower intracellular levels of GSH in the LoVo cells.

*In vitro* studies with tumour cells in this laboratory have investigated the involvement of GSH in protection against a range of cytotoxic agents. We observed that the cytotoxicities of menadione, helenalin and melphalan were potentiated due to GSH depletion by BSO, however the effects of BSO were relatively modest (Table I). It is of particular interest to compare these results with other studies in the literature to assess the potential use of BSO in chemosensitising tumour cells (Table II). BSO has been reported to increase drug toxicity, sensitise drug resistant cell lines and also reduce drug toxicity. The majority of effects are clearly very modest and some results actually conflict, possibly due to different effects in different cell lines. Under *in vitro* conditions, it is possible to choose a concentration and time period of BSO exposure which would cause optimal GSH depletion, prior to incubation with the cytotoxic chemicals. If, under such optimum conditions, only a slight potentiation of toxicity is observed, it is difficult to envisage any great potentiation of toxicity to the tumour tissue occurring *in vivo,* especially as it will be necessary to consider other important factors such as the pharmacokinetics of BSO and the antitumour agent. Furthermore GSH depletion *in vivo* due to administration of BSO will not be confined to tumour tissue therefore potentiation of toxicity to normal tissue may be a limiting factor as was recently observed with the enhanced nephrotoxicity of rats treated with BSO (Kramer *et al.*, 1985).

Our results and those of others suggest that great caution should be exercised in the potential use of BSO in the chemosensitisation of tumours in man.

This work was supported in part by the Cancer Research Campaign of Great Britain. We are grateful to Dr B. Drewinko for permission to utilise the LoVo cells, kindly supplied by Dr B. Hill (Imperial Cancer Research Fund). We thank Mrs M. Fagg for the typing of the manuscript and Mr D. King for the preparation of figures.

**References**

AKMAN, S.A., DIETRICH, M., CHLEBOWSKI, R., LIMBERG, P. & BLOCK, J.B. (1985). Modulation of cytotoxicity of menadione sodium bisulfite *versus* Leukaemia L1210 by the acid soluble thiol pool. *Cancer Res.*, **45**, 5257.

ARRICK, B.A., NATHAN, C.F. & COHN, Z.A. (1983). Inhibition of glutathione synthesis augments the lysis of murine tumour cells by sulphydryl-reactive antineoplastics. *J. Clin. Invest.*, **71**, 258.

ARRICK, B.A., NATHAN, C.F., GRIFFITH, O.N. & COHN, Z.A. (1982). Glutathione depletion sensitizes tumour cells to oxidative cytolysis. *J. Biol. Chem.*, **257**, 1231.

BACHUR, N., GORDON, S.L. & GEE, M.W. (1978). General mechanism for microsomal activation of quinone anticancer agents to free radicals. *Cancer Res.*, **38**, 1745.

BRODIE, A.E. & REED, D.J. (1985). Buthionine sulphoximine inhibition of cystine uptake and glutathione biosynthesis in human lung carcinoma cells. *Toxicol. Appl. Pharmac.*, **77**, 381.

CAPRANICO, G., BABUDRI, N., CASCIARRI, G. & 6 others (1986). Lack of effect of glutathione on cytotoxicity, mutagenicity and DNA damage, produced by doxorubicin in cultured cells. *Chem. Biol. Interact.*, **57**, 189.

CHLEBOWSKI, R.T., BLOCK, J.B., DIETRICH, M., OCTAY, E., BARTH, N., YANAGIHARA, R., GOTA, C. & 5 others (1983). Inhibition of human tumour growth and DNA biosynthetic activity by vitamin K and warfarin: *in vitro* and clinical results. *Proc. Am. Assoc. Cancer Res.*, **24**, 653.

COHEN, G.M., WILSON, G.D., GIBBY, E.M., SMITH, M.T., D'ARCY DOHERTY, M. & CONNORS, T.A. (1983). 1-Naphthol: A potential selective antitumour agent. *Biochem. Pharmacol.*, **33**, 2363.

D'ARCY DOHERTY, M. & COHEN, G.M. (1984*a*). Metabolic activation of 1-naphthol by rat liver microsomes to 1,4-naphthoquinone and covalent binding species. *Biochem. Pharmacol.*, **33**, 543.

D'ARCY DOHERTY, M., COHEN, G.M. & SMITH, M.T. (1984*b*). Mechanisms of toxic injury to isolated hepatocytes by 1-naphthol. *Biochem. Pharmacol.*, **33**, 543.

D'ARCY DOHERTY, M., MAKOWSKI, R., GIBSON, G.G. & COHEN, G.M. (1985). Cytochrome P-450 dependent metabolic activation of 1-naphthol to naphthoquinones and covalent binding species. *Biochem. Pharmacol.*, **34**, 2261.

DEGRAFF, W.G., RUSSO, A. & MITCHELL, J.B. (1985). Glutathione depletion greatly reduces NCS cytotoxicity in Chinese hamster V79 cells. *J. Biol. Chem.*, **260**, 8512.

DETHMERS, J.K. & MEISTER, A. (1981). Glutathione export by human lymphoid cells: Depletion of GSH by inhibition of its synthesis decreases export and increases sensitivity to irradiation. *Proc. Natl Acad. Sci, USA*, **78**, 7492.

DREWINKO, B., ROMSDAHL, M.M., YANG, L.-Y., AHEARN, M.J. & TRUJILLO, J.M. (1976). Establishment of a human carcinoma antigen producing colonic adenocarcinoma cell line. *Cancer Res.*, 3, 467.

GREEN, J.A., VISTICA, D.T., YOUNG, R.C., HAMILTON, T.C., ROGAN, A.M. & OZOLS, R.F. (1984). Potentiation of melphalan cytotoxicity in human ovarian cancer cell lines by glutathione depletion. *Cancer Res.*, **44**, 5427.

GRIFFITH, O.W. & MEISTER, A. (1979). Potent and specific inhibition of glutathione synthesis by buthionine sulphoximine (S-n-Butyl Homocysteine Sulphoximine). *J. Biol. Chem.*, **254**, 7558.

HALL, I.H., LEE, K.H. & EIGEBALLY, S.A. (1977). Effects of helanalin on anaerobic and aerobic metabolism of Ehrlich ascites tumour cells. *J. Pharm. Sci.*, **67**, 553.

HALL, I.H., LEE, K.H., MAR, E.C. & STARNES, O.C. (1977). A proposed mechanism for inhibition of cancer growth by tenulin and helenalin and related cyclopentenones. *J. Med. Chem.*, **20**, 333.

HAMILTON, T.C., WINTER, M.A., LOUIE, K.J. & 7 others (1985). Augmentation of adriamycin, melphalan and *cis*-platin cytotoxicity in drug resistant and sensitive human ovarian carcinoma cell lines by BSO mediated GSH depletion. *Biochem. Pharmacol.*, **34**, 2583.

HISSIN, P.J. & HILF, R. (1976). A fluorometric method for determination of oxidised and reduced glutathione in tissues. *Anal. Biochem.*, **74**, 214.

JONES, M., DOHERTY, M. D'ARCY & COHEN, G.M. (1987). Antitumour activity of 1-naphthol against L1210 leukaemia *in vivo* and Ehrlich ascites tumour cells *in vivo* and *in vitro*. *Cancer Lett.*, **33**, 347.

KAPPUS, H., & SIES, H. (1981). Toxic drug effects associated with oxygen metabolism: Redox cycling and lipid peroxidation. *Experientia*, **37**, 1233.

KRAMER, R.A., SCHULLER, H.M., SMITH, A.C. & BOYD, M.R. (1985). Effects of buthionine sulfoximine on the nephrotoxicity of 1-(2-chlooethyl)-3-(Trans-4-methylcyclohexyl)1-1nitrosourea (MeCCNU), *J. Pharmacol. Exp. Ther.*, **234**, 498.

MEISTER, A. & ANDERSON, M. (1979). Glutathione. *Ann. Rev. Biochem.*, **52**, 711.

NICKERSON, W.J., FALCONE, G. & STRAUSS, G. (1963). Studies on quinone thioethers. I. Mechanism of formation and properties of thiodione. *Biochemistry*, **2**, 537.

POWIS, G., SVINGEN, B.A. & APPEL, P. (1981). Quinone stimulated superoxide formation by subcellular fractions in isolated hepatocytes and other cells. *Mol. Pharmacol,*, **20**, 387.

ROMINE, M.T. & KESSEL, D. (1986). Intracellular glutathione as a determinant of responsiveness to antitumour drugs. *Biochem. Pharmacol.*, **35**, 3323.

RUSSO, A., DEGRAFF, W., FRIEDMANN, N. & MITCHELL, J.B. (1986). Selective modification of GSH levels in human normal *versus* tumour cells and subsequent differential response to chemotherapy. *Cancer Res.*, **46**, 2845.

SMITH, M.T., EVANS, C.G., THOR, H. & ORRENIUS, S. (1985). Quinone induced oxidative injury to cells and tissues. In *Oxidative Stress*, Sies, H. (ed) p. 91. Academic Press: New York.

SOMFAI-RELLE, S., SUZUKAKE, K., VISTICA, B.P. & VISTICA, D.T. (1984). Reduction in cellular glutathione by BSO and sensitization of murine tumour cells resistant to L-phenylalanine mustard. *Biochem. Pharmacol.*, **33**, 485.

SUZUKAKE, K., PETRO, B.J. & VISTICA, D.T. (1982). Reduction in GSH content of L-PAM resistant L1210 cells confers drug sensitivity. *Biochem. Pharmacol.*, **31**, 121.

SUZUKAKE, K., VISTICA, B.P. & VISTICA, D.T. (1983). Dechlorination of L-PAM by sensitive and resistant tumour cells and its relationship to intracellular GSH content. *Biochem. Pharmacol.*, **32**, 165.

THOR, H., SMITH, M.T., HARTZELL, P., BELLOMO, G., JEWELL, S.A. & ORRENIUS, S. (1982). Metabolism of menadione (2-methyl-1,4-naphthoquinone) by isolated hepatocytes. *J. Biol. Chem.*, **257**, 12419.

TSAN, M.F., DANIS, E.H., DEL VECCHIO, P.J. & ROSONO, C.L. (1985). Enhancement of intracellular glutathione protects endothelial cells against oxidant damage. *Biochem. Biophys. Res. Comm.*, **127**, 270.

WENDEL, A. (1981). Glutathione peroxidase, In *Enzymatic Basis of Detoxification*, Jakoby, W.B. (ed) p. 325. Academic Press: New York.

WILSON, G.D., D'ARCY DOHERTY, M. & COHEN, G.M. (1985). Selective toxicity of 1-naphthol to human colorectal tumour tissue. *Br. J. Cancer*, **51**, 853.


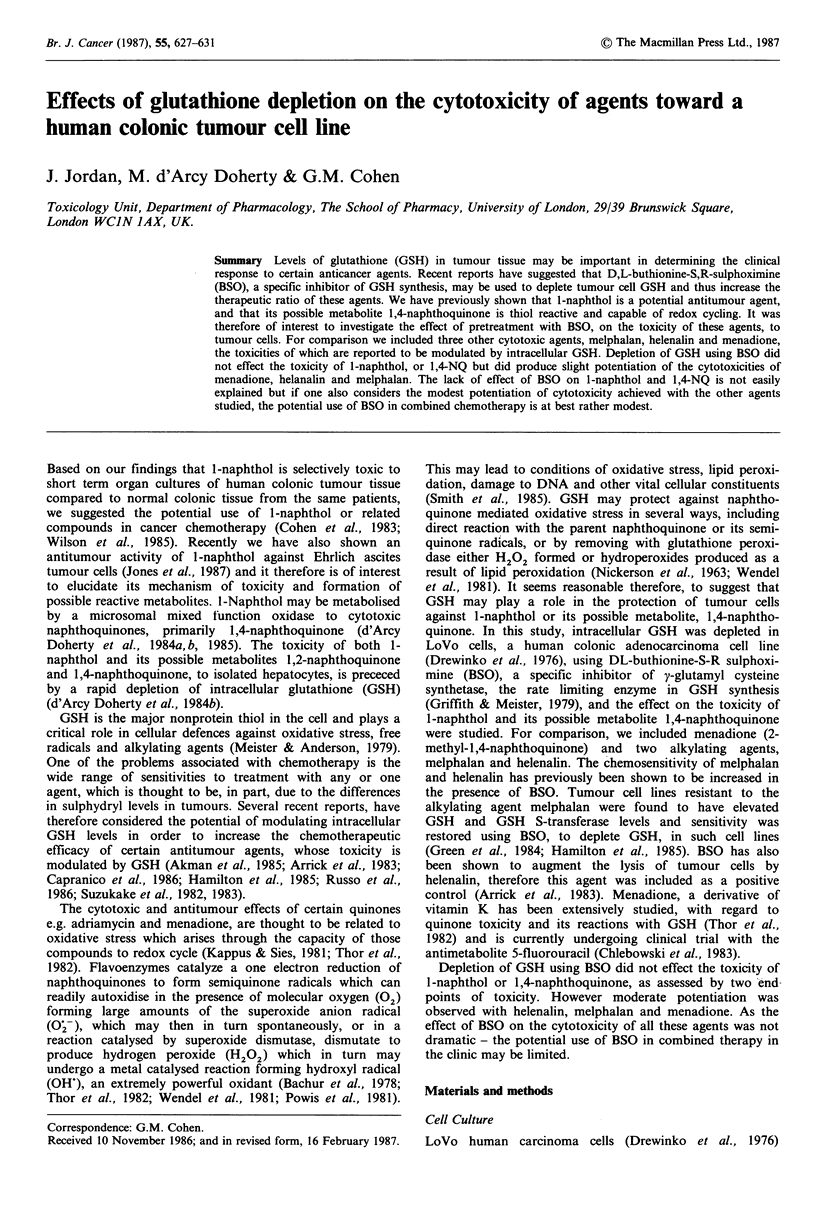

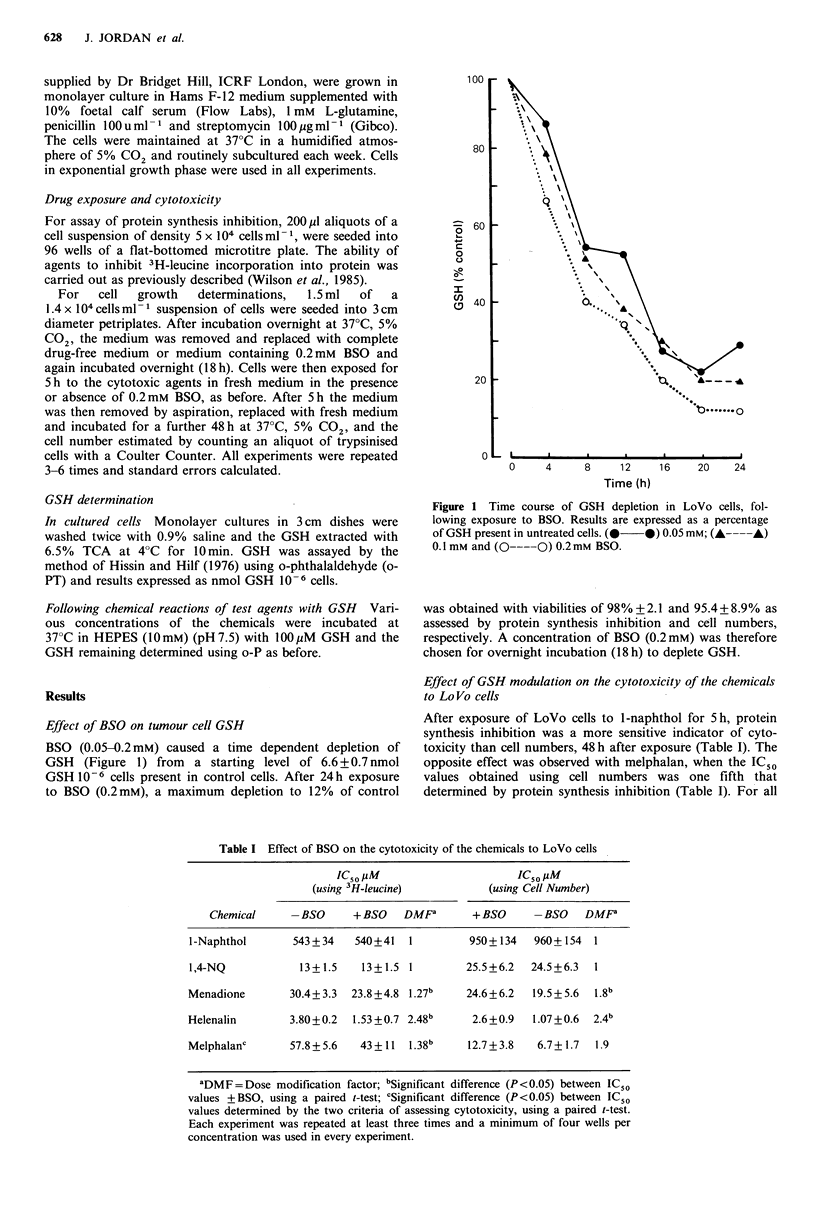

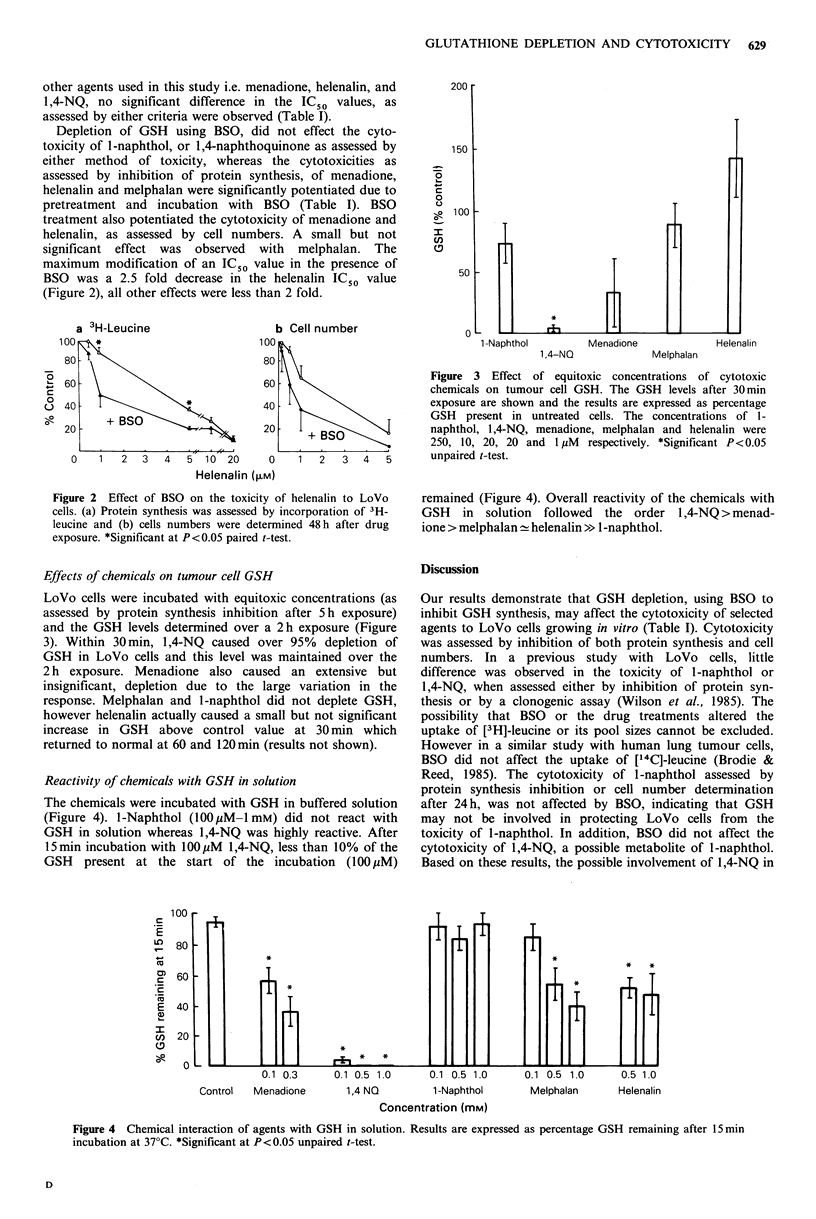

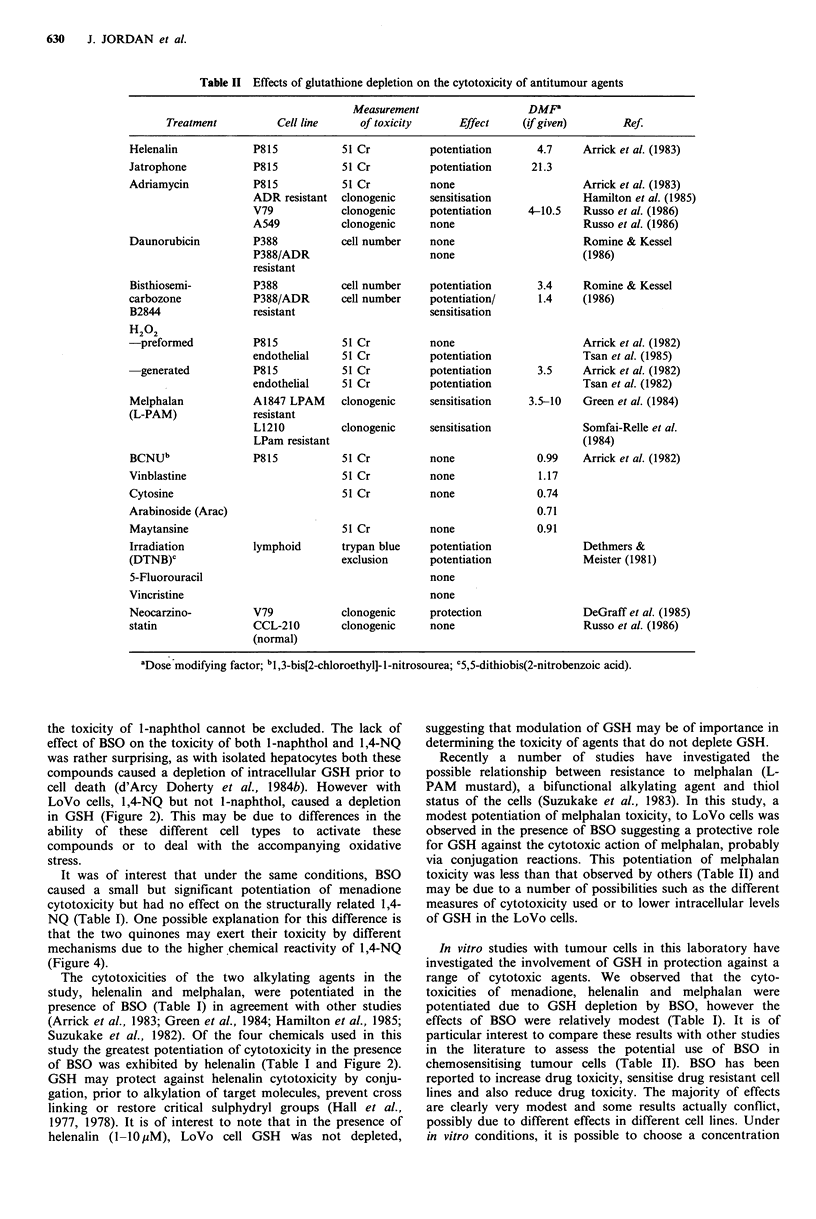

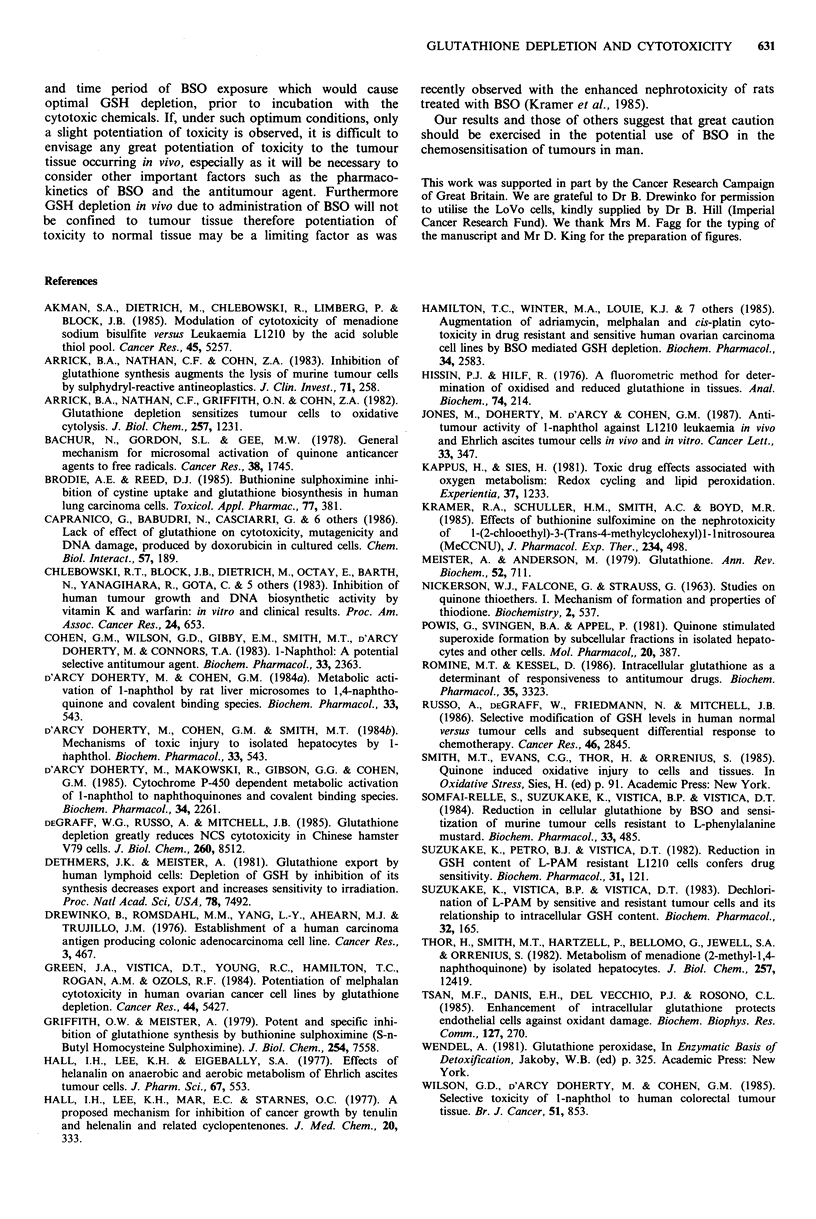


## References

[OCR_00665] Akman S. A., Dietrich M., Chlebowski R., Limberg P., Block J. B. (1985). Modulation of cytotoxicity of menadione sodium bisulfite versus leukemia L1210 by the acid-soluble thiol pool.. Cancer Res.

[OCR_00671] Arrick B. A., Nathan C. F., Cohn Z. A. (1983). Inhibition of glutathione synthesis augments lysis of murine tumor cells by sulfhydryl-reactive antineoplastics.. J Clin Invest.

[OCR_00676] Arrick B. A., Nathan C. F., Griffith O. W., Cohn Z. A. (1982). Glutathione depletion sensitizes tumor cells to oxidative cytolysis.. J Biol Chem.

[OCR_00681] Bachur N. R., Gordon S. L., Gee M. V. (1978). A general mechanism for microsomal activation of quinone anticancer agents to free radicals.. Cancer Res.

[OCR_00686] Brodie A. E., Reed D. J. (1985). Buthionine sulfoximine inhibition of cystine uptake and glutathione biosynthesis in human lung carcinoma cells.. Toxicol Appl Pharmacol.

[OCR_00691] Capranico G., Babudri N., Casciarri G., Dolzani L., Gambetta R. A., Longoni E., Pani B., Soranzo C., Zunino F. (1986). Lack of effect of glutathione depletion on cytotoxicity, mutagenicity and DNA damage produced by doxorubicin in cultured cells.. Chem Biol Interact.

[OCR_00706] Cohen G. M., Wilson G. D., Gibby E. M., Smith M. T., Doherty M. D., Connors T. A. (1983). 1-Naphthol: a potential selective antitumour agent.. Biochem Pharmacol.

[OCR_00731] Dethmers J. K., Meister A. (1981). Glutathione export by human lymphoid cells: depletion of glutathione by inhibition of its synthesis decreases export and increases sensitivity to irradiation.. Proc Natl Acad Sci U S A.

[OCR_00720] Doherty M. A., Makowski R., Gibson G. G., Cohen G. M. (1985). Cytochrome P-450 dependent metabolic activation of 1-naphthol to naphthoquinones and covalent binding species.. Biochem Pharmacol.

[OCR_00711] Doherty M. D., Cohen G. M., Smith M. T. (1984). Mechanisms of toxic injury to isolated hepatocytes by 1-naphthol.. Biochem Pharmacol.

[OCR_00737] Drewinko B., Romsdahl M. M., Yang L. Y., Ahearn M. J., Trujillo J. M. (1976). Establishment of a human carcinoembryonic antigen-producing colon adenocarcinoma cell line.. Cancer Res.

[OCR_00743] Green J. A., Vistica D. T., Young R. C., Hamilton T. C., Rogan A. M., Ozols R. F. (1984). Potentiation of melphalan cytotoxicity in human ovarian cancer cell lines by glutathione depletion.. Cancer Res.

[OCR_00749] Griffith O. W., Meister A. (1979). Potent and specific inhibition of glutathione synthesis by buthionine sulfoximine (S-n-butyl homocysteine sulfoximine).. J Biol Chem.

[OCR_00759] Hall I. H., Lee K. H., Mar E. C., Starnes C. O., Waddell T. G. (1977). Antitumor agents. 21. A proposed mechanism for inhibition of cancer growth by tenulin and helenalin and related cyclopentenones.. J Med Chem.

[OCR_00765] Hamilton T. C., Winker M. A., Louie K. G., Batist G., Behrens B. C., Tsuruo T., Grotzinger K. R., McKoy W. M., Young R. C., Ozols R. F. (1985). Augmentation of adriamycin, melphalan, and cisplatin cytotoxicity in drug-resistant and -sensitive human ovarian carcinoma cell lines by buthionine sulfoximine mediated glutathione depletion.. Biochem Pharmacol.

[OCR_00772] Hissin P. J., Hilf R. (1976). A fluorometric method for determination of oxidized and reduced glutathione in tissues.. Anal Biochem.

[OCR_00777] Jones M., d'Arcy Doherty M., Cohen G. M. (1986). Antitumour activity of 1-naphthol against L1210 leukaemia in vivo and Ehrlich ascites tumour cells in vivo and in vitro.. Cancer Lett.

[OCR_00783] Kappus H., Sies H. (1981). Toxic drug effects associated with oxygen metabolism: redox cycling and lipid peroxidation.. Experientia.

[OCR_00788] Kramer R. A., Schuller H. M., Smith A. C., Boyd M. R. (1985). Effects of buthionine sulfoximine on the nephrotoxicity of 1-(2-chloroethyl)-3-(trans-4-methylcyclohexyl)-1-nitrosourea (MeCCNU).. J Pharmacol Exp Ther.

[OCR_00794] Meister A., Anderson M. E. (1983). Glutathione.. Annu Rev Biochem.

[OCR_00798] NICKERSON W. J., FALCONE G., STRAUSS G. (1963). STUDIES ON QUINONE-THIOETHERS. I. MECHANISM OF FORMATION AND PROPERTIES OF THIODIONE.. Biochemistry.

[OCR_00803] Powis G., Svingen B. A., Appel P. (1981). Quinone-stimulated superoxide formation by subcellular fractions, isolated hepatocytes, and other cells.. Mol Pharmacol.

[OCR_00808] Romine M. T., Kessel D. (1986). Intracellular glutathione as a determinant of responsiveness to antitumor drugs.. Biochem Pharmacol.

[OCR_00813] Russo A., DeGraff W., Friedman N., Mitchell J. B. (1986). Selective modulation of glutathione levels in human normal versus tumor cells and subsequent differential response to chemotherapy drugs.. Cancer Res.

[OCR_00824] Somfai-Relle S., Suzukake K., Vistica B. P., Vistica D. T. (1984). Reduction in cellular glutathione by buthionine sulfoximine and sensitization of murine tumor cells resistant to L-phenylalanine mustard.. Biochem Pharmacol.

[OCR_00830] Suzukake K., Petro B. J., Vistica D. T. (1982). Reduction in glutathione content of L-PAM resistant L1210 Cells confers drug sensitivity.. Biochem Pharmacol.

[OCR_00835] Suzukake K., Vistica B. P., Vistica D. T. (1983). Dechlorination of L-phenylalanine mustard by sensitive and resistant tumor cells and its relationship to intracellular glutathione content.. Biochem Pharmacol.

[OCR_00841] Thor H., Smith M. T., Hartzell P., Bellomo G., Jewell S. A., Orrenius S. (1982). The metabolism of menadione (2-methyl-1,4-naphthoquinone) by isolated hepatocytes. A study of the implications of oxidative stress in intact cells.. J Biol Chem.

[OCR_00847] Tsan M. F., Danis E. H., Del Vecchio P. J., Rosano C. L. (1985). Enhancement of intracellular glutathione protects endothelial cells against oxidant damage.. Biochem Biophys Res Commun.

[OCR_00858] Wilson G. D., d'Arcy Doherty M., Cohen G. M. (1985). Selective toxicity of 1-naphthol to human colorectal tumour tissue.. Br J Cancer.

